# Protective effect of higher free thyroxine levels within the reference range on biliary tract cancer risk: a multivariable mendelian randomization and mediation analysis

**DOI:** 10.3389/fendo.2024.1379607

**Published:** 2024-04-15

**Authors:** Yuxian Chen, Hao Dong, Baozhen Qu, Xuezhen Ma, LinLin Lu

**Affiliations:** ^1^ College of Medicine, Qingdao University, Qingdao, China; ^2^ Qingdao Cancer Prevention and Treatment Research Institute, Qingdao Central Hospital, University of Health and Rehabilitation Sciences (Qingdao Central Hospital), Qingdao, China; ^3^ Department of Oncology, Qingdao Central Hospital, University of Health and Rehabilitation Sciences (Qingdao Central Hospital), Qingdao, China

**Keywords:** thyroid function, hepatobiliary cancer, free thyroxine, metabolic syndrome, waist circumference, Mendelian randomization

## Abstract

**Background:**

Hepatobiliary cancer (HBC), including hepatocellular carcinoma (HCC) and biliary tract cancer (BTC), is currently one of the malignant tumors that mainly cause human death. Many HBCs are diagnosed in the late stage, which increases the disease burden, indicating that effective prevention strategies and identification of risk factors are urgent. Many studies have reported the role of thyroid hormones on HBC. Our research aims to assess the causal effects and investigate the mediation effects between thyroid function and HBC.

**Methods:**

Utilizing the Mendelian randomization (MR) approach, the study employs single nucleotide polymorphisms (SNPs) as instrumental variables (IVs) to explore causal links between thyroid function [free thyroxine (FT4), thyroid stimulating hormone (TSH), hyperthyroidism and hypothyroidism] and HBC. Data were sourced from the ThyroidOmic consortium and FinnGen consortium. The analysis included univariable and multivariable MR analysis, followed by mediation analysis.

**Results:**

The study found a significant causal association between high FT4 levels and the reduced risk of BTC, but not HCC. However, TSH, hyperthyroidism and hypothyroidism had no causal associations with the risk of HBC. Notably, we also demonstrated that only higher FT4 levels with the reference range (FT4-RR) could reduce the risk of BTC because this protective effect no longer existed under the conditions of hyperthyroidism or hypothyroidism. Finally, we found that the protective effect of FT4-RR on BTC was mediated partially by decreasing the risk of metabolic syndrome (MetS) and reducing the waist circumference (WC).

**Conclusion:**

The findings suggest that higher FT4-RR may have a protective effect against BTC, which is partially mediated by decreased risk of MetS and a reduction in WC. This study highlights the potential role of FT4 in the pathogenesis of BTC and underscores that MetS and WC may play mediation effects as two mediators in this process.

## 1 Introduction

Hepatobiliary cancer (HBC), including hepatocellular carcinoma (HCC) and biliary tract cancer (BTC), is currently the sixth most common cancers worldwide in terms of incidence (1). Despite advancements in both localized and systemic treatments of HBC in recent years, the 5-year survival rate remains below 20% in HCC and 5% in BTC (2, 3). Relevant statistical data show that the incidence and mortality of HBC will continually rise in the next several decades (4). Moreover, many HBCs are diagnosed at advanced stages, which exacerbates the disease burden, indicating that effective prevention strategies and identification of risk factors are urgent for the prevention of HBC. Several risk factors, including alcohol consumption, viral hepatitis, and non-alcoholic fatty liver disease (NAFLD), have been identified as important triggers for the development of HCC (5) In BTC, metabolism, obesity-related characteristics and hepatobiliary disease are the common risk factors (6). However, there are still around 20% of HCC patients and 50% of BTC patients are diagnosed without any identifiable risk factors (6, 7). Therefore, it is necessary to identify new potential risk factors and take appropriate preventive measures to cope with the increasing incidence rate of HBC.

Thyroid gland is an endocrine organ that secretes thyroid hormones (THs) to regulate numerous physiological and pathophysiological processes via THs receptors including thyroid hormone receptor α (TRα) and β (TRβ), playing a key role in affecting cell differentiation, proliferation, autophagy, and metabolic processes (8, 9). THs, including triiodothyronine (T3) and thyroxine (T4), are very conserved hormones that play crucial roles in development and the regulation of cellular metabolism, cell structure and membrane transport (10). THs regulate physiological and pathophysiological processes by affecting gene expressions through interactions with nuclear receptors (long-term effects/genomic effects) and also by activating protein kinases and/or ion channels (short-term effects). THs play a core role in regulating glucose, lipid, and cholesterol metabolism, therefore THs disorders may lead to severe pathological conditions. In addition, metabolism is closely related to cancer, and by-products of metabolism can promote oncogenic DNA mutations (11). TH metabolites such as diiodothyronine (T2), monoiodothyronine (T1) and thyronine (T0) also have been demonstrated to possess significant biological effects (12). For example, both 3,5-diiodothyronine (3,5-T2) and 3,3’- diiodothyronine (3,3’-T2) could increase the oxidation rate of rat mitochondria by activating cytochrome c oxidase activity (13). Moreover, a calorigenic effect of 3,5-T2 and 3,3’-T2 was found in rats (14). Another study (15) demonstrated that in hypothyroid rats, 3,5-T2 could enhance mitochondrial respiration, activate thermogenesis, increase sympathetic innervation and vascularization in brown adipose tissue (BAT). 3,5-T2 could also regulate physiological processes in the liver, such as activating F_0_F_1_-ATP synthase in liver mitochondria and reducing lipid synthesis in liver cells (16, 17). Moreover, 3-iodothyronamine (3-T1AM) and thyronamine (T0AM) also exhibited biologically activities in metabolism regulation, cardiac and brain function (12).

There is a lot of evidence to suggest that THs can lead to the physiological and pathological response of liver cancer. For example, T3/TR interaction could inhibit Wnt/β-catenin pathway through Dickkopf Wnt signaling inhibitor 4 (DKK4), thereby inhibiting the proliferation of liver cancer cells (18). Another study found that T3 could inhibit the growth of liver cancer cells via increasing the G1 phase of the cell cycle, which is related to the decreased expression of cell cycle mediators cyclin-dependent kinase 2 and cyclin E, as well as the increased expression of transforming growth factor TGF-β (19). Moreover, it was reported that TRβ1 could act as an anti-metastatic factor to inhibit the activation of ERK and PI3K pathways, thereby inhibiting the nuclear signal transduction pathway of HCC (20–22). In the past decade, the complex association between THs and cancer development has attracted increasing attention. Recent research indicated that thyroid dysfunction was related to the risks of various cancers including breast, prostate, ovarian, and colon cancers (23–26). However, although several studies revealed the association between THs and HCC, it is difficult to determine the causal association between thyroid dysfunction and HCC, and the impact of thyroid function on the pathogenesis of HCC remains controversial. A case-control study demonstrated a significant association between hypothyroidism and increased HCC risk [odds ratio (OR) = 2.9], while history of hyperthyroidism showed no significant relation to HCC (OR = 1.7) after adjusting for known risk factors (27). Another study revealed that 20% of HCC patients with initial euthyroid function developed hypothyroidism during treatment, indicating that the progression or treatment of HCC may interfere with thyroid function (28). Moreover, the association between thyroid function and the risk of BTC has rarely been reported. BTC includes gallbladder cancer and cholangiocarcinoma, most patients are diagnosed at an advanced stage (29), and the 5-year survival rate is around 5% (30). Although viral hepatitis, cholelithiasis, cholangitis and bile duct cysts are the common risk factors for BTC, there are still around 50% of cases are diagnosed without any identifiable risk factors (6) in Western countries. Therefore, understanding the underlying causes and risk factors of this disease is imperative.

Mendelian randomization (MR) is a novel epidemiological approach utilizing single nucleotide polymorphisms (SNPs) as instrumental variables (IVs) to ascertain causative links between risk factors and disease outcomes. The core theory of MR is based on Mendel’s second law, which states that alleles of different genes assort independently of one another during gametogenesis. MR is considered a natural simulation of a randomized controlled trial. Moreover, the genotype is independent of confounding elements including socioeconomic status, environment and behavior, so MR strengthens the validity of causal inferences by reducing potential biases. Lu et al. employed MR methodology to identify a negative association between hypothyroidism and the risk of HCC (31). However, in Lu et al.’s study, the sample for the GWAS of hypothyroidism and HCC both comes from the UK Biobank (UKB). It implied that the GWAS data for exposure and outcomes were derived from the same samples (MR analysis should be conducted between two independent databases), making sample overlap and affecting the results of their MR analysis. (32). Besides, there are no studies analyzing the association between thyroid function and BTC. With the recent availability of FinnGen data freeze10, the newest GWAS database contains a BTC and HCC database respectively and has been updated until December 2023. We recalibrated the correlation between THs and HCC/BTC separately using FinnGen data freeze 10. In summary, through the latest and most comprehensive database and rigorous MR analysis, we have reached a conclusion that FT4 levels positively correlate with lower incidence of BTC by being probably involved in reducing risks of metabolic syndrome (MetS) and decreasing waist circumference (WC).

## 2 Methods

**Figure 1:**
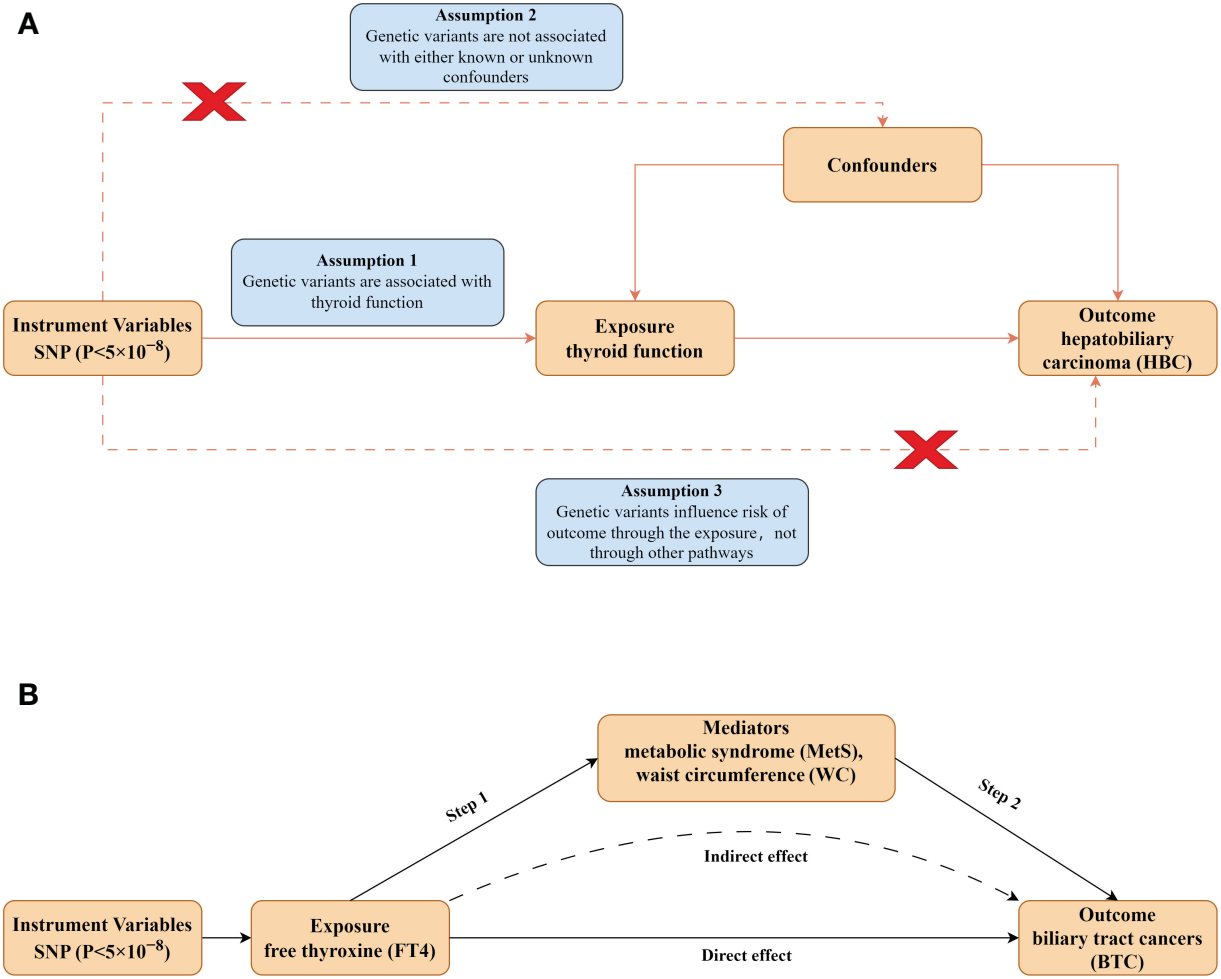
Design of the present study. **(A)** Schematic diagram of MR analysis satisfying three key assumptions. SNP, Single nucleotide polymorphism. **(B)** Two-step MR analysis framework. Step 1 estimated the causal effect of the exposure on the potential mediators, and step 2 assessed the causal effect of the mediators on BTC risk.

MR analysis provides an effective method for examining the causal effects of exposure on disease development, utilizing genetic variation as the instrumental variable (IV) (33, 34). This approach reduces the impact of unmeasured confounding factors, thereby enhancing the precision of causal inferences (35). We selected two independent databases ThyroidOmic consortium and FinnGen consortium from the European population and employed a univariable MR to investigate the causative connections between thyroid function (TSH, FT4 levels, hyperthyroidism and hypothyroidism) and HBC. Then, we performed multivariate MR (MVMR) analysis to exclude the influence of the pathological status of hyperthyroidism and hypothyroidism. Finally, we carried out a mediation analysis of MR to explore the mediating pathways between thyroid function and HBC development. The MR design is predicated on three core assumptions (**Figure 1A**) (1): the genetic variants are strongly associated with thyroid function (2): the genetic variants are not associated with any confounders related to HBC prognosis (3): the genetic variants influence only HBC through their effect on thyroid function (36, 37). To scrutinize the causal association between thyroid function and HBC, a bidirectional MR analysis was conducted, adhering to the Strengthening the Reporting of Observational Studies in Epidemiology-Mendelian Randomization (STROBE-MR) principles (38). Given the public availability of the genetic data utilized, ethical approval was deemed unnecessary for this study. A detailed flowchart summarized the study design (**Figure 1B**).

### 2.1 Data source

We retrieved the GWAS thyroid function summary data from the ThyroidOmic consortium, which collected the most extensive collection of thyroid phenotype-related GWAS (39). TSH data were collated from 22 independent cohorts, encompassing 54288 subjects, while data on FT4 (49269 subjects), hyperthyroidism (1840 cases and 49983 controls), and hypothyroidism (3340 cases and 49983 controls) were acquired from 19 independent cohorts. Subjects with non-European ancestry, taking thyroid drugs, or undergoing thyroid surgery were excluded. To avoid sample overlap and ensure genetic profile similarity between exposure and outcome datasets, data on HBC patients were sourced from data freeze10 (R10) of the FinnGen consortium (40). FinnGen facilitates the exploration of genetic variation and disease patterns in isolated populations, providing data on HCC and BTC. The total sample size for this version was 412181 participants, with 21311942 variants examined.

### 2.2 Selection of genetic instruments

Before MR analysis, SNPs underwent rigorous screening to ascertain quality. SNPs exhibiting strong associations with exposure at a genome-wide significance threshold (P < 5 × 10^-8^) were retained, and their genes were evaluated using linkage disequilibrium (LD) clustering against European ancestry reference data. To ensure independence between included SNPs, linkage was established (r^2 ^= 0.001, clustering window=10000 kb). The F-statistic was then calculated for the selected SNPs, excluding those with F statistics below 10 to mitigate the impact of weak instrumental variables on the final results. A comprehensive review of HBC risk factors from existing literature was conducted, and each selected SNP was examined on PhenoScanner to eliminate factors associated with potential confounders. Finally, SNPs directly associated with HBC were excluded by setting a genome-wide significance threshold (P > 5 × 10^-5^), satisfying the third hypothesis that genetic variation affects the outcome only through risk factors.

### 2.3 Univariable MR analysis and sensitivity analysis

Inverse Variance Weighting (IVW) serves as the primary analytical method for estimating causal effects, operating under the assumption that all SNPs are valid IVs and are mutually independent. Complementary to IVW, MR-Egger and Weighted Median (WM) methods were also employed to enhance the robustness of MR analysis (41). When heterogeneity existed, we used the multiplicative random effects IVW method for MR analysis (42). The presence of horizontal pleiotropy was evaluated using the MR-Egger intercept test, and a leave-one-out analysis was conducted to determine the influence of individual SNPs on the overall results (43). In this analysis, one genetic variation was selected as the test set and the remaining k-1 genetic variations as the training set, allowing for the assessment of whether the cumulative results were driven by a genetic variant exhibiting high levels of pluripotency. Variations in results, pre and post the removal of genetic variation, indicating sensitivity to genetic variation. To investigate potential confounding factors that might influence the direction of the causal effect, we selected outcome as exposure and exposure as the outcome for reverse MR analysis.

Heterogeneity among IVs served as a marker for potential violations of the IV assumption. Cochran’s Q test was employed to quantify the heterogeneity of IVW, with significant heterogeneity defined as P < 0.05, following the χ^2^ distribution (44). All analysis were conducted utilizing ‘TwoSampleMR’ package (version 0.5.6) and R software (version 4.2.1), with the significance level set at a p-value of 0.05 (two-sided). We conducted MR Steiger directionality tests to ensure reliable directional analysis (45). In addition, we used causal analysis with summary effect estimates (CAUSE) to assess whether the association between exposure and outcome was affected by correlational level pleiotropy (variants affecting outcome and exposure through shared genetic factors) (46).

### 2.4 Multivariable MR analysis

MVMR analysis is a method that allows for the association of SNPs with multiple phenotypes to be included in the analysis, allowing an estimation of the direct effect of each phenotype on the outcome. To further evaluate whether the causal effects of FT4 on BTC were affected by hyperthyroidism or hypothyroidism, we conducted MVMR analysis. Considering that there might be overlapping or correlated SNPs in composite IVs (the sum of IVs from different traits or exposures), we thus removed SNPs in linkage disequilibrium (r^2^ > 0.001) to obtain a list of independent SNPs, by applying the ‘clump_data’ function (r^2 ^= 0.001, clustering window=10000 kb) of the ‘TwoSampleMR’ package (version 0.5.6).

### 2.5 Mediation analysis

We further performed a mediation analysis using a two-step MR design to explore whether risk factors associated with thyroid function play a potential mediating role. As with the univariate MR analysis, LD clustering was used (r^2 ^= 0.001, clustering window = 10000 kb) and data that were significantly associated with exposure at the genome-wide significance threshold (P < 5 × 10^-8^) were retained. The overall effect can be decomposed into an indirect effect (through mediators) and a direct effect (without mediators). The total effect of thyroid function on hepatobiliary carcinoma was decomposed into (1) direct effects of thyroid function on hepatobiliary carcinoma and (2) indirect effects mediated by thyroid function through the mediator. We calculated the percentage mediated by the mediating effect by dividing the indirect effect by the total effect. Meanwhile, 95% confidence interval (CI) was calculated with the delta method.

## 3 Results

### 3.1 Univariable MR

GWAS data of thyroid function and HBC were harmonized, and SNPs were identified for the MR analysis, including 15 SNPs for hyperthyroidism, 10 SNPs for hypothyroidism, 41 SNPs for TSH, 21 SNPs for FT4 (**Supplementary Tables 1**-**9**), which explained 33.03%, 14.26%, 7.27%, and 3.41% of the variance of these risk factors, respectively (**Supplementary Tables 10**, **11**). The F-statistic values of the remaining SNPs indicated no potential weak IV bias, with values ranging from 29.91 to 935.59.

**Figure 2:**
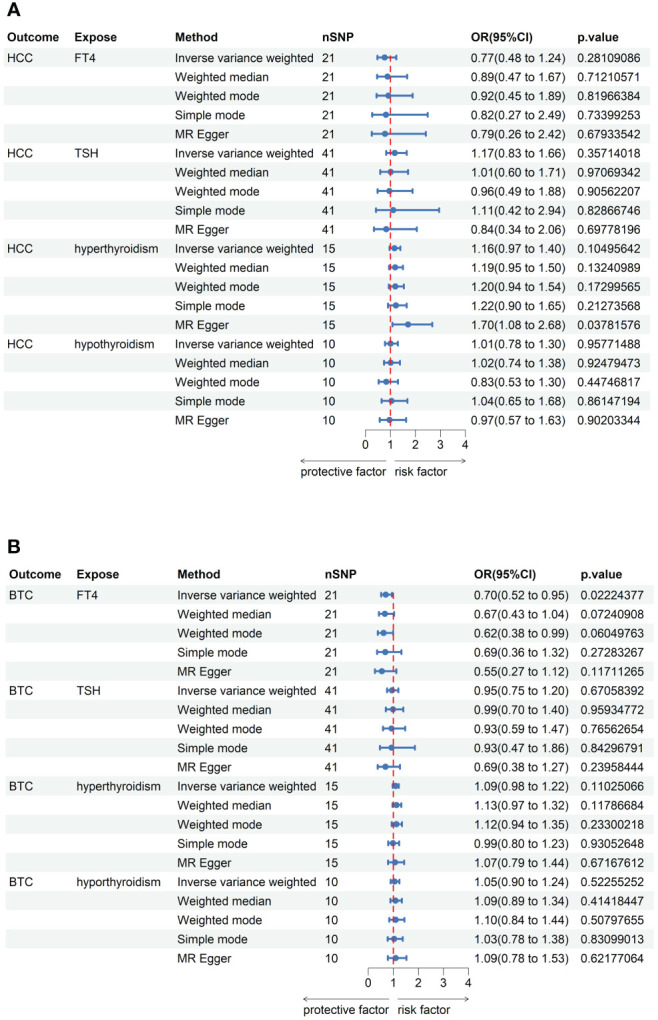
MR analysis results between thyroid function and risk of hepatobiliary carcinoma. **(A)** The forest plot existing causal effect of thyroid function on HCC. **(B)** The forest plot existing causal effect of thyroid function on BTC.

**Figure 3:**
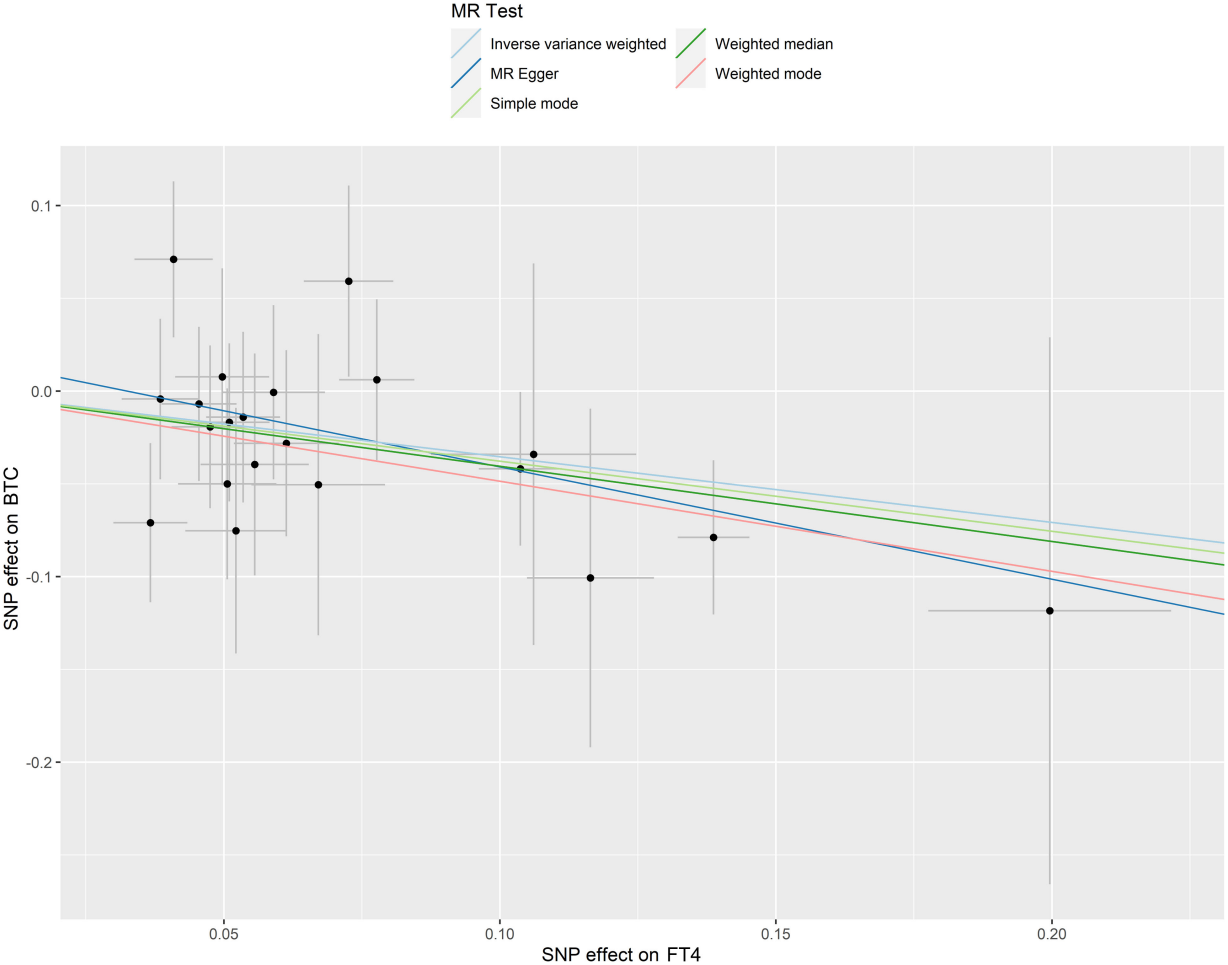
Scatter plots of SNP effects on FT4 level and BTC risk. X axes represent SNP effects on FT4 level. Y axes represent SNP effects on BTC risk.

No outlier or horizontal pleiotropy was detected for any association, after conducting the MR-PRESSO test and intercept test (all P > 0.05, **Table 1**). A significant causal association was identified between FT4 level and risk of BTC using the IVW method (OR = 0.70, 95% CI = 0.52-0.95, P = 0.02, **Table 2**), but no such association was found between FT4 and HCC (**Figures 2A, B**). A scatterplot of the association between FT4 level and risk of BTC was shown in **Figure 3**, with the colored lines representing the slopes of different regression analysis. Bayesian CAUSE analysis suggested that the causal model is a better fit to data than the sharing model (delta_elpd = -1.07 < 0) (**Supplementary Figure 1**). Additionally, no evidence was found for a causal effect of genetically predicted hyperthyroidism, hypothyroidism, or TSH level on the risk of BTC or risk of HCC (**Supplementary Figures 2**, **3**). Sensitivity analysis revealed no heterogeneity in these four datasets (**Table 1**). The robustness of our results was confirmed through leave-one-out analysis, as no SNPs with a significant dominant effect on the results were identified. Furthermore, analysis missing each SNP showed that no single SNP drove these results (**Supplementary Figures 4**, **5**). We found no evidence of reverse causality in the MR Steiger test (**Supplementary Table 12**).

**Table 1:** Details of GWAS data and results of sensitivity analysis.

Exposure	Exposure data source	Ancestry	Outcome	Outcome data source	Cases	Sample size	Ancestry	n-SNPs	P-heterogeneity	P-intercept
Hyperthyroidism	ThyroidOmics consortium	European	HCC	FinnGen consortium	500	412181	European	15	0.32	0.06
Hypothyroidism								11	0.95	0.97
TSH								41	0.52	0.45
FT4								21	0.96	0.80
Hyperthyroidism	ThyroidOmics consortium	European	BTC	FinnGen consortium	1207	412181	European	15	0.61	0.92
Hypothyroidism								11	0.83	0.85
TSH								41	0.22	0.32
FT4								21	0.94	0.50
HCC	FinnGen consortium	European	Hyperthyroidism	ThyroidOmics consortium	3545	462933	European	51	0.42	0.24
			Hypothyroidism		51194	494577		52	0.16	0.69
			TSH		NA	3301		51	0.89	0.47
			FT4		NA	26231		52	0.94	0.22
BTC	FinnGen consortium	European	Hyperthyroidism	ThyroidOmics consortium	1840	49983	European	60	0.16	0.11
			Hypothyroidism		3340	49983		63	0.24	0.99
			TSH		NA	54288		61	0.17	0.73
			FT4		NA	49269		63	0.21	0.32

n-SNPs, number of single nucleotide polymorphisms; P-heterogeneity, P-value of heterogeneity test; P-intercept, P-value of intercept test; TSH, thyroid stimulating hormone; FT4, free thyroxine; HCC, hepatocellular carcinoma; BTC, biliary tract cancer.

NA, not applicable.

**Table 2:** Multivariable MR analysis estimating the FT4 on BTC, conditioning on hyperthyroidism or hyperthyroidism.

Model	OR (95% CI)	SE	*P*	F	Q
Without adjustment					
FT4 without adjustment	0.70 (0.52, 0.95)	0.15	0.02	78.31	9.66
After adjustment					
FT4 adjusted for hyperthyroidism	1.01 (0.75, 1.34)	0.15	0.99	59.19	48.42
FT4 adjusted for hyporthyroidism	1.02 (0.77, 1.37)	0.15	0.87	59.19	29.61

OR, odds ratio; SE, standard error; P, P-value of the MR effect estimate; F, instrument strength; Q, Cochran’s Q heterogeneity statistic.

### 3.2 Reverse MR analysis

To assess potential confounding factors that might influence the direction of the causal effect, we selected HBC as exposure and thyroid function as the outcome for reverse MR. No evidence was found to suggest a potential causal association between genetically predicted HBC and thyroid function (**Table 1**, **Supplementary Tables 13**, **14**).

### 3.3 Multivariable MR

Considering that FT4 levels are interrelated with hyperthyroidism and hypothyroidism, we performed MVMR to estimate the direct effect of FT4 levels on the risk of BTC under hyperthyroidism and hypothyroidism conditions respectively. IVs used for MVMR analysis are listed in **Supplementary Table 15**. Notably, the effect of FT4 levels on the risk of BTC lost causality in MVMR after accounting for hyperthyroidism and hypothyroidism. This result suggested that the protective effect of FT4 levels against BTC was affected under conditions of hyperthyroidism or hypothyroidism. The MVMR estimates for hyperthyroidism (IVW OR = 0.56, 95% CI = 0.16-1.96, P = 0.37) and hypothyroidism (IVW OR = 0.56, 95% CI = 0.20-1.55, P = 0.27) were not statistically significant (**Table 2**).

### 3.4 Mediation analysis

Metabolism, obesity-related characteristics and hepatobiliary disease risk reduction may be mediators of the protective effects of relative THs against BTC. We performed a two-step MR analysis by analyzing MetS, body mass index (BMI), WC, cholecystitis, cholelithiasis, biliary cirrhosis, NAFLD, primary biliary cholangitis (PBC) and primary sclerosing cholangitis (PSC) related phenotypes to investigate the mediating pathway from FT4 levels to BTC. In the first step, IVs at the FT4 level are used to estimate the causal effect of exposure on the underlying mediator. Among 9 potential mediators, we found only a causal association between WC, MetS and FT4 levels, with increased FT4 levels associated with lower WC (IVW β = -0.026, 95% CI = -0.050 to -0.003, P = 0.025) and lower risk of MetS (IVW multiplicative random effects OR = 0.957, 95% CI = 0.919 to 0.997, P = 0.037) (**Supplementary Table 16**). In a second step, we assessed the causal effect of the mediator on BTC risk using IVs for the causal mediator-related phenotype. We found causal evidence for the impact of MetS (IVW OR = 1.223, 95% CI = 1.019 to 1.467, P = 0.03) and WC (IVW OR = 1.753, 95% CI = 1.233 to 2.492, P = 0.002) on BTC.

Finally, we estimated the indirect effect of FT4 level on the risk of BTC via mediators and found that the mediation effect of MetS was -0.008, with a mediated proportion of 2.4%, and the mediation effect of WC was -0.016, with a mediated proportion of 4.4% (**Table 3**).

**Table 3:** The mediation effect of FT4 level on risk of BTC via MetS or WC.

Exposure	Mediator	Outcome	Total effect^a^			Direct effect α^b^			Direct effect β^c^			Mediation effect^d^	
			Beta^e^	SE	*P*	Beta^e^	SE	*P*	Beta^e^	SE	*P*	Effect^f^	Proportion
FT4	MetS	BTC	-0.354	0.155	0.022	-0.044	0.021	0.037	0.192	0.089	0.031	-0.008	2.40%
	WC					-0.026	0.012	0.025	0.593	0.173	0.001	-0.016	4.40%

SE, standard error; P, P-value of the MR effect estimate; ^a^The causal effect of FT4 level on risk of BTC in MR analysis, ^b^The causal effect of FT4 on mediation in MR analysis, ^c^The causal effect of Mediation on BTC in analysis, ^d^The effect of FT4 level on risk of BTC through Mediation, ^e^Beta of random effect inverse variance weighted method was used for MR analysis, ^f^mediation effect was derived by using the delta method.

## 4 Discussion

MR analysis is a methodological approach utilizing genetic diversity to explore potential causal associations between risk factors and disease outcomes. We used MR analysis to clarify that genetically predicted hyperthyroidism, hypothyroidism and TSH have no causal association with the risk of HCC and BTC. Surprisingly, we observed evidence indicating the protective effects of genetically predicted FT4 levels on BTC risk but not on HCC, and this causal association no longer existed after accounting for hyperthyroidism and hypothyroidism. Furthermore, we conducted a mediation analysis to estimate potential mediators and showed that MetS and WC played mediating roles in the association between FT4 and BTC. In all, this discovery revealed the protective role of FT4 in the progression of BTC, providing evidence for future inclusion of thyroid hormone profiles into individual patient cancer risk assessments.

Thyroid function plays an important role in regulating physiological and pathophysiological processes. Research in animal models and human studies indicated that thyroid hormone regulated cellular processes associated with age-related diseases such as diabetes, cardiovascular complications, neurocognitive dysfunction, and cancer (47, 48). In clinical practice, thyroid function is monitored by measuring circulating TSH and free FT4 levels, with elevated TSH indication of hypothyroidism and low TSH indication of hyperthyroidism. Therefore, in this study, we selected FT4 and TSH levels within the reference range as well as hyperthyroidism and hypothyroidism as exposure factors representing thyroid function for MR analysis. In previous research, clinical findings supported an increased risk of hepatocellular carcinoma with hypothyroidism, suggesting that TH abnormalities might be a risk factor for this cancer. Reddy et al. reported that after adjusting for confounding factors, HCC patients with unknown cancer etiology had a significantly increased OR of 12.7 (95% CI = 1.4-117.1) for hypothyroidism compared with HCC patients with alcoholic liver disease or hepatitis C (49). In another report, patients with hypothyroidism had twice the risk of developing HCC compared with patients without thyroid disease (28). This association was particularly significant among female patients, even when the analysis was adjusted for sex as a covariate. In addition, a study reported that high TSH level was related to larger tumor size but not survival when adjusted for known prognostic factors for HCC (50). However, in our study results, thyroid function was not related to the risk of HCC. The reason why our research results differ from the above clinical research results may be due to the interference of many confounding factors (i.e. obesity, diabetes and NAFLD) in these observational clinical studies, resulting in difficulty in establishing the causal association between thyroid function and HCC (51–53). In contrast, the MR research method using SNP as IVs can well control the interference of confounding factors to obtain more reliable results. However, the specific underlying mechanism still needs further studies.

Compared to HCC, the association between BTC and thyroid function has received far less attention in scientific research. While BTC does share some risk factors with HCC, BTC lacks its own key risk marker, making it necessary to study the two separately. To our knowledge, this is the first study on the causal association between thyroid function and BTC risk. We found that FT4 is a protective factor for BTC (OR = 0.70, 95% CI = 0.52-0.95, P = 0.02), and confirmed no reverse causation through reverse analysis. In addition, MR-PRESSO analysis, MR-Egger regression analysis, and MR-Egger test showed that heterogeneous pleiotropy had no impact on the results, and no single SNP affected the overall MR estimate. However, after adjusting for hyperthyroidism and hypothyroidism as confounders, this protective effect was no longer present, which suggested that FT4 provided protection only under normal thyroid function. Observational studies have reached different conclusions on the association between FT4 levels and cancers. The Rotterdam study showed that after excluding subjects using thyroid function-altering medications, higher FT4 levels were associated with a 1.13-fold increased risk of any solid cancer (24). Another retrospective cohort study based on Clalit Health Services (CHS) by Krashin et al. noted that the effect of FT4 on cancer risks varied by age and malignancy type (54). In patients younger than 50 years, elevated FT4 was associated with increased overall cancer risks [(hazard ratio) HR = 1.28, 95% CI = 1.1-49.4], whereas in patients 50 years or older with hyperthyroidism (FT4 > 1.55 ng/dL) had a lower cancer risk (adjusted HR = 0.87, 95% CI = 0.76-0.99). Across tumor types, elevated FT4 was associated with an increased risk of lung cancer (adjusted HR = 1.54, 95% CI = 1.1-2.03) and a significantly reduced risk of colorectal cancer (HR = 0.59, 95% CI = 0.41-0.85). As far as the specific role of FT4 in BTC, further clinical studies are needed to elucidate the potential role of thyroid function in the pathogenesis of BTC.

There are two different ways that THs (i.e. T3 and T4) regulate the physiological and pathological processes. On the one hand, the long-term effects of THs on gene expressions are regulated by thyroid hormone receptors (TRs), which belong to the nuclear hormone receptor superfamily, mediating homeostatic control of almost all biological processes such as development, reproduction, cell growth, metabolism, immunity and inflammation (12). On the other hand, the short-term effects of THs mediates several cellular functions mainly through integrin αvβ3, which is one of the transmembrane adhesion receptors belonging to the integrin family. These THs short-term effects include the support of liver fibrosis (55), the proliferation of airway smooth muscle cells (56), the expansion of neural progenitor cells in the brain cortex and the development of normal brain (57). In addition, integrin αvβ3-mediated effects of the THs related to cancers have also been extensively studied. Pro-survival activity of THs initiated through short-term effects has been reported in malignant T cell lymphoma cells (58), differentiated and undifferentiated pheochromocytoma cells (59) and mesenchymal stem cells (MSCs) studied in hepatoma cell-conditioned environment (60). In the process of cancer development, the short-term effects mainly occur at the plasma membrane level and involve membrane transport systems including the transporters for glucose and amino acids, the Na^+^/K^+^-ATPase activity, the Na^+^/H^+^ exchanger, and kinase activities such as Mitogen-Activated Protein Kinase (MAPK) and Phosphatidyl Inositol 3 Kinase (PI3K), therefore increasing angiogenesis and the growth of tumor cells (18).

At last, we conducted a two-step MR for mediation analysis and showed that the protective effect of higher FT4-RR on BTC risk was partially mediated by decreased risk of MetS and a reduction in WC. In the first MR step, univariate MR determined the causal association between FT4 and MetS risk, as well as WC. The second step of MR analysis provided evidence that genetically determined MetS and WC are inversely associated with BTC. An analysis from the Surveillance, Epidemiology and End Results (SEER) reported that patients with MetS had a 1.56-fold increased risk of developing intrahepatic cholangiocarcinoma (OR = 1.56, 95% CI = 1.32-1.83, P < 0.0001) (61). Another case-control study showed that MetS was positively associated with cholangiocarcinoma in the Chinese patient population (OR = 1.86, 95% CI = 1.29-2.66, P = 0.001) (62). The influence of metabolic factors on cancer is receiving increasing attention, and the association between hepatobiliary carcinoma and MetS is expected to become a major cause of disease in Western countries in the next few years (63, 64). In addition, WC is often used to assess central adiposity, which has been shown to increase the risk of several cancer types (65, 66). In this study, we found that FT4 reduces BTC risk by lowering WC. To our knowledge, this is the first MR analysis to demonstrate that MetS and WC increased the risk of BTC. There are currently limited studies investigating the association between MetS, WC and BTC, and observational prospective studies are needed to clarify this point.

This study is the first to establish a causal association between thyroid function and BTC through MR analysis, minimizing confounding factors and reverse associations. Nevertheless, this study is not devoid of limitations. The reliance on GWAS data based on European ancestry to circumvent population stratification bias limited the generalizability of our results to other ethnic groups. Additionally, the absence of stratified GWAS for BTC and gender-related data restricted our stratified analysis. Despite these limitations and the divergence in results between IVW and other MR analysis methods, the adherence to basic MR assumptions and the meticulous removal of potential outliers prior to analysis bolsters the reliability of our findings.

## 5 Conclusion

In conclusion, this is the first MR analysis on the association between thyroid function and HBC risk. This two-sample MR study genetically predicted higher FT4-RR as the protective factor of BTC but not HCC in the absence of hyperthyroidism and hypothyroidism. Furthermore, this protective effect of FT4 on BTC was mediated partly by decreasing the risk of MetS and a reduction in WC. In addition, we also demonstrated that hyperthyroidism, hypothyroidism and TSH have no causal association with the risk of HBC. These findings emphasize the potential for the inclusion of FT4 level in future BTC risk prediction models. However, future randomized clinical trials will be still needed to confirm this genetic inference.

## Data availability statement

The original contributions presented in the study are included in the article/**Supplementary Material**. Further inquiries can be directed to the corresponding authors.

## Author contributions

YC: Conceptualization, Writing – original draft. HD: Methodology, Writing – original draft. BQ: Conceptualization, Writing – original draft. XM: Supervision, Visualization, Writing – review & editing. LL: Project administration, Writing – review & editing.
