# Supplementary material for: Protective effect of higher free thyroxine levels within the reference range on biliary tract cancer risk: a multivariable mendelian randomization and mediation analysis
Source: Front Endocrinol (Lausanne). 2024 Apr 15;15:1379607. doi: 10.3389/fendo.2024.1379607 (PMC11056546; doi:10.3389/fendo.2024.1379607)
Supplement: Supplementary file 1 [file DataSheet_1.docx]

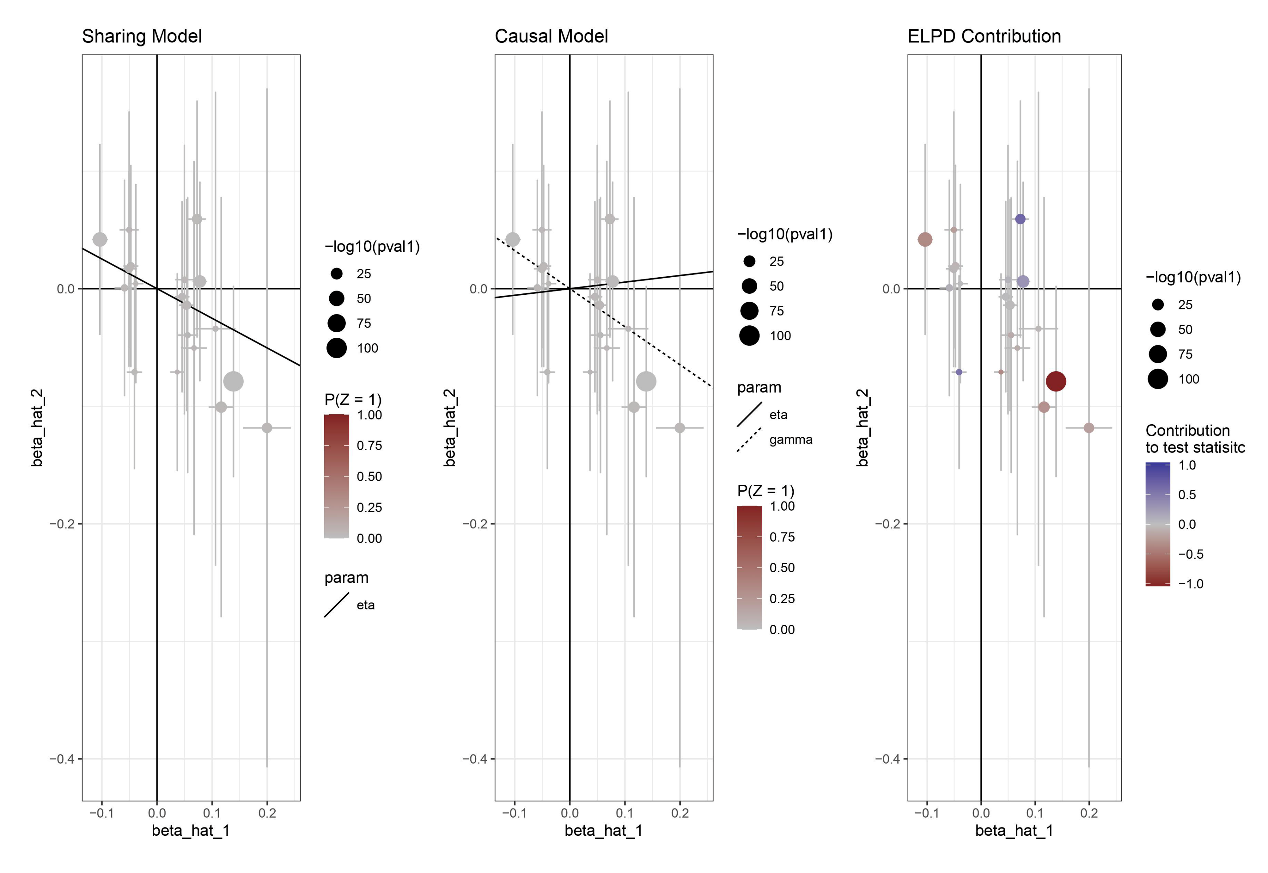


**Supplementary Figure 1.** CAUSE analysis for the genetically predicted effect of FT4 on BTC. The ELPD contribution plot visually represents the contribution of each SNP to the test statistic. The plot shows only genome-wide significant SNPs. Warmer tones indicate a contribution to the causal model, while colder tones indicate a contribution to the sharing model.


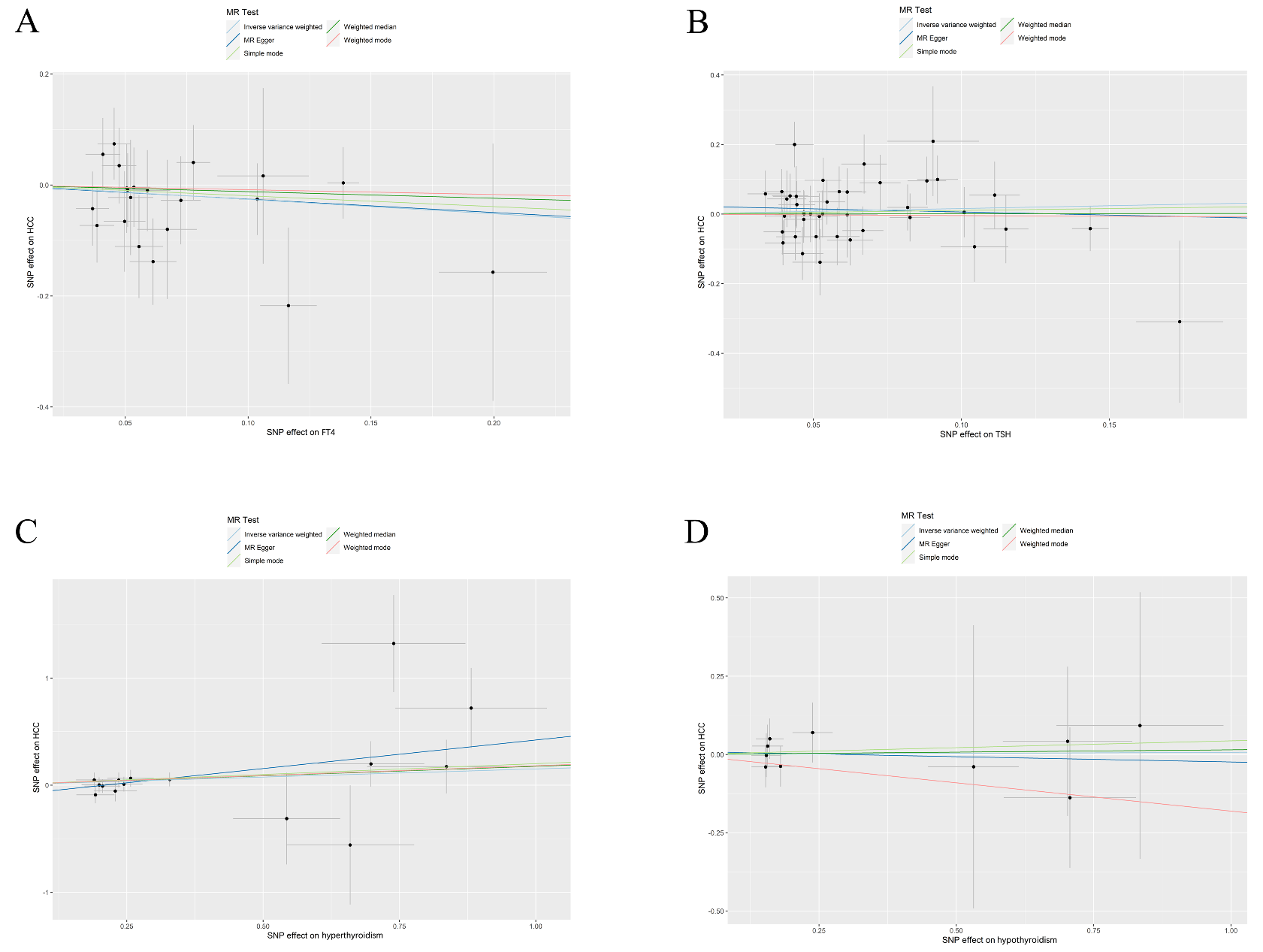


**Supplementary Figure 2.** Scatter plots of SNP effects on thyroid function and HCC risk. **(A)** X axes represent SNP effects on FT4 level. Y axes represent SNP effects on HCC risk. **(B)** X axes represent SNP effects on TSH level. Y axes represent SNP effects on HCC risk. **(C)** X axes represent SNP effects on hyperthyroidism. Y axes represent SNP effects on HCC risk. **(D)** X axes represent SNP effects on hypothyroidism. Y axes represent SNP effects on HCC risk.


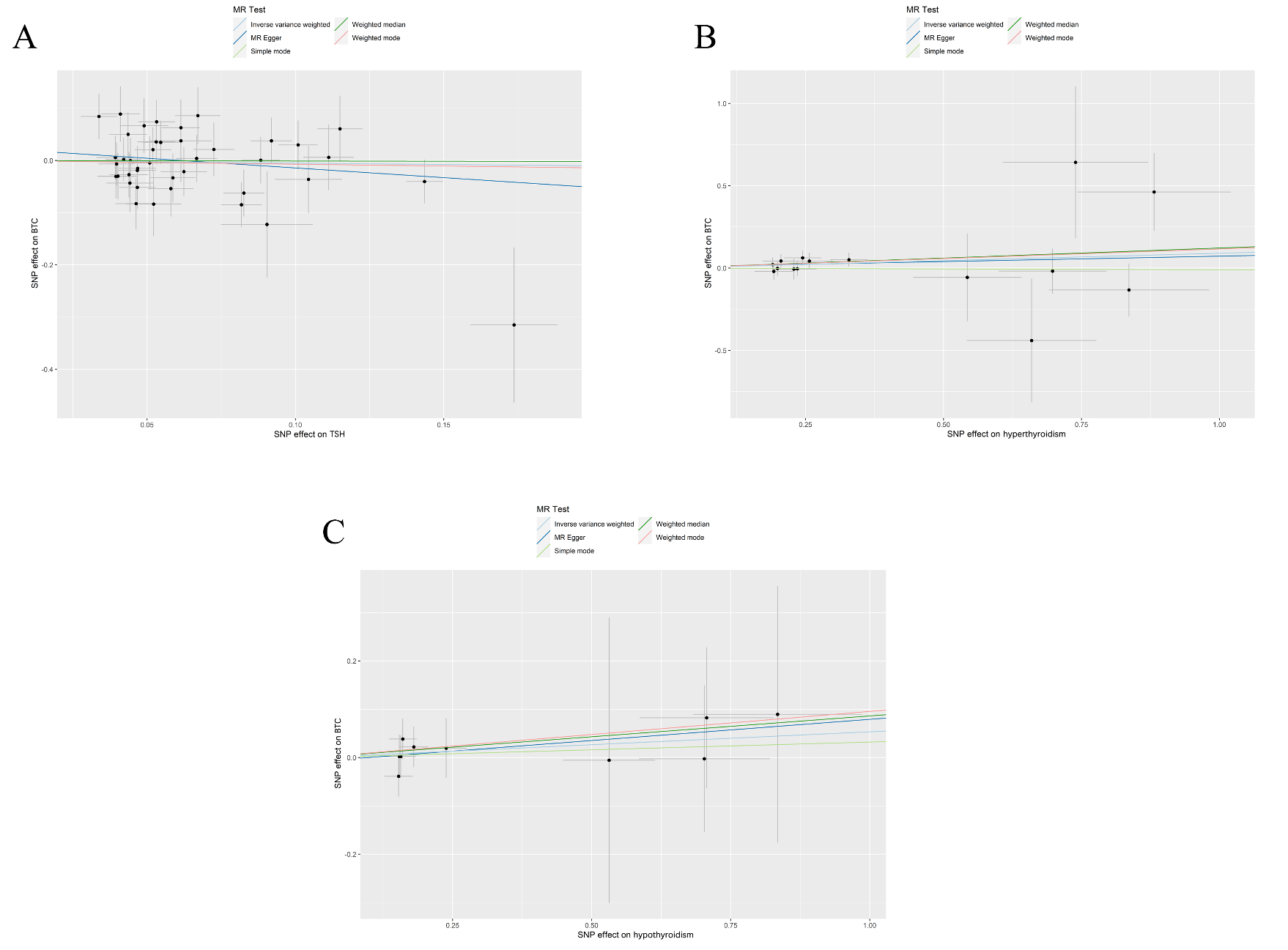


**Supplementary Figure 3.** Scatter plots of SNP effects on thyroid function and BTC risk. **(A)** X axes represent SNP effects on TSH level. Y axes represent SNP effects on BTC risk. **(B)** X axes represent SNP effects on hyperthyroidism. Y axes represent SNP effects on BTC risk. **(C)** X axes represent SNP effects on hypothyroidism. Y axes represent SNP effects on BTC risk.


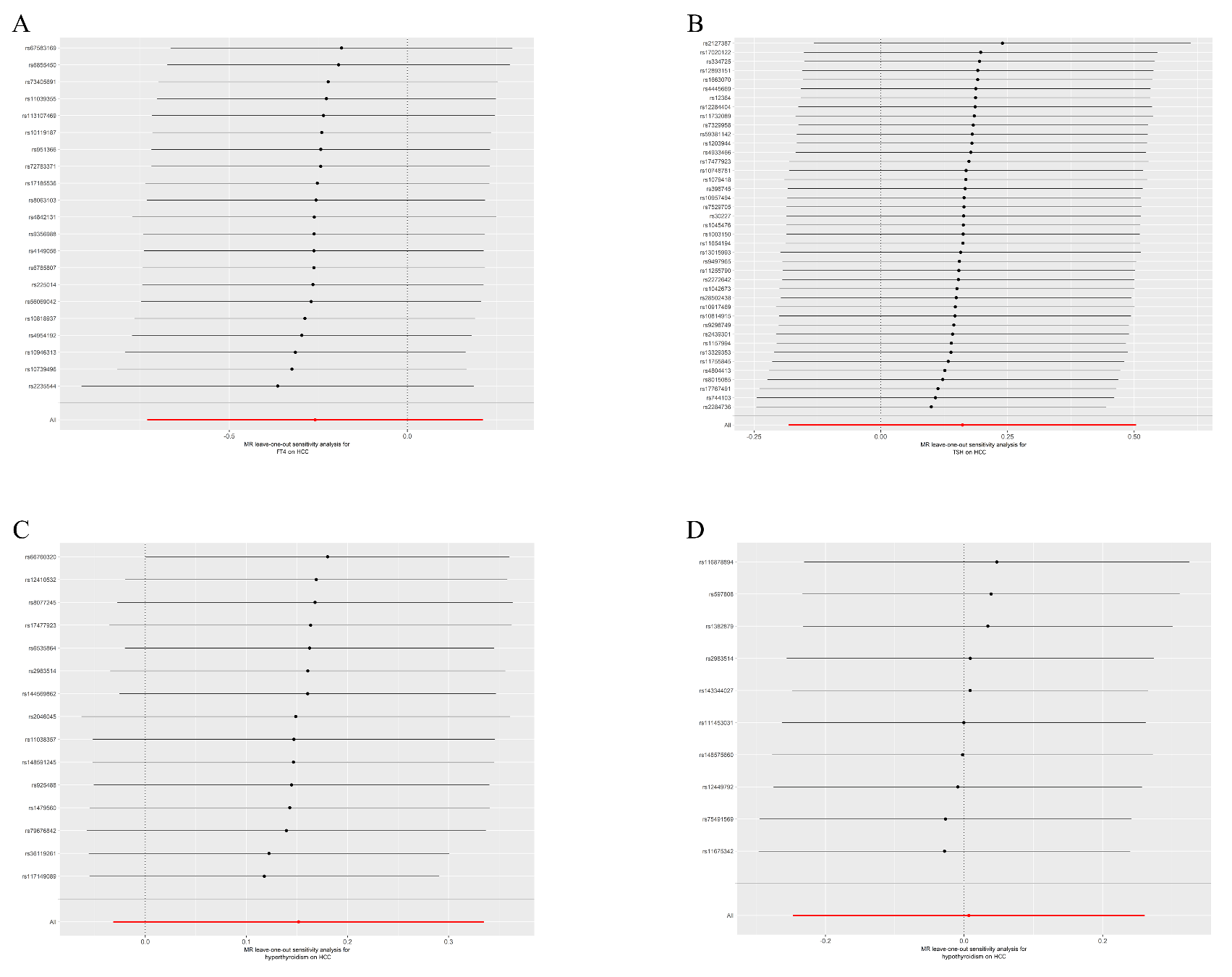


**Supplementary Figure 4.** Leave-one-out analyses of thyroid function and HCC risk MR results. **(A)** Leave-one-out analyses of FT4 level and HCC risk MR results. **(B)** Leave-one-out analyses of TSH level and HCC risk MR results. **(C)** Leave-one-out analyses of hyperthyroidism and HCC risk MR results. **(D)** Leave-one-out analyses of hypothyroidism and HCC risk MR results.


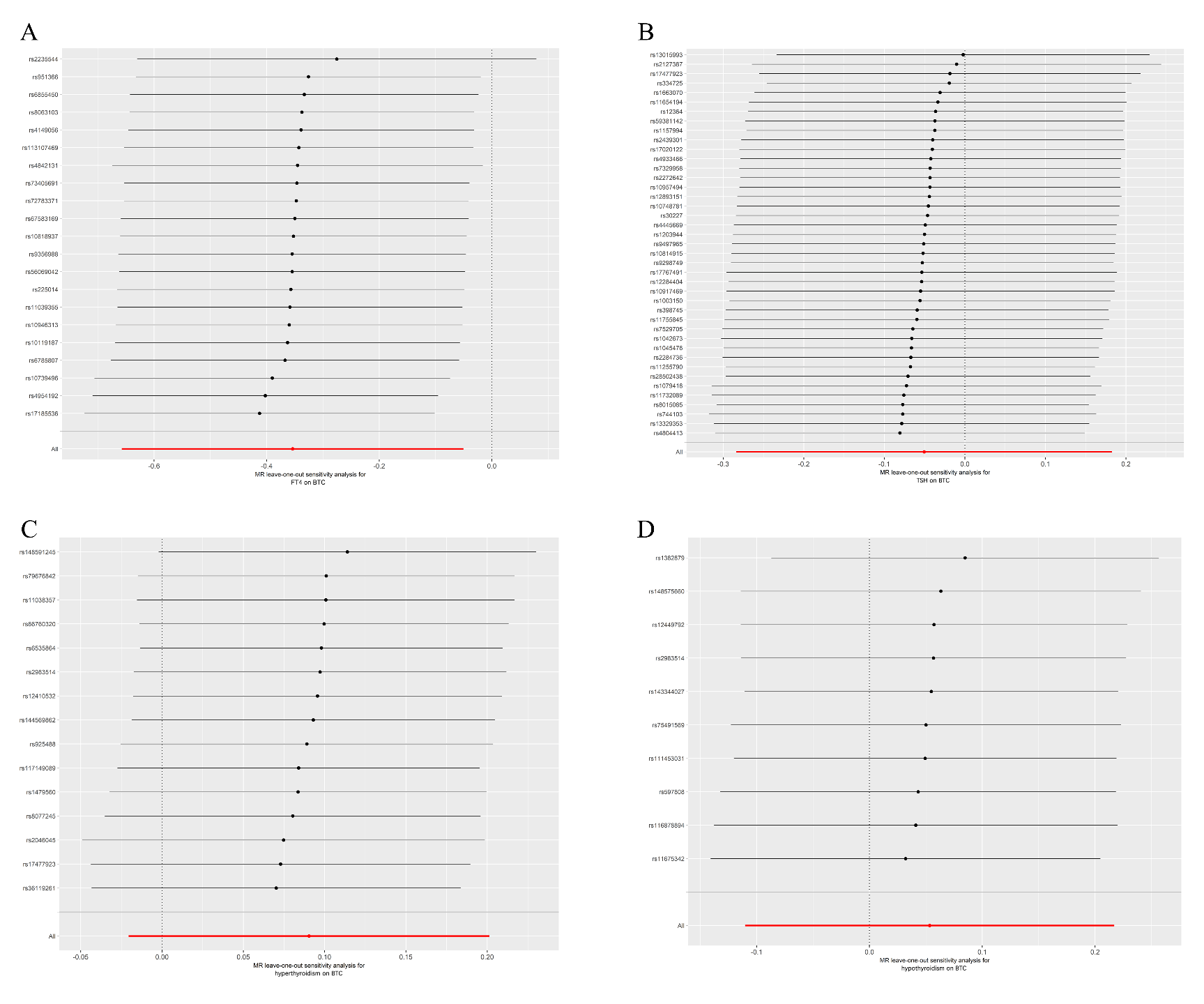


**Supplementary Figure 5.** Leave-one-out analyses of thyroid function and BTC risk MR results. **(A)** Leave-one-out analyses of FT4 level and BTC risk MR results. **(B)** Leave-one-out analyses of TSH level and BTC risk MR results. **(C)** Leave-one-out analyses of hyperthyroidism and BTC risk MR results. **(D)** Leave-one-out analyses of hypothyroidism and BTC risk MR results.
